# Circulating Heat Shock Protein 60 Levels Are Elevated in HIV Patients and Are Reduced by Anti-Retroviral Therapy

**DOI:** 10.1371/journal.pone.0045291

**Published:** 2012-09-28

**Authors:** Itaru Anraku, Reena Rajasuriar, Caroline Dobbin, Richard Brown, Sharon R. Lewin, Andreas Suhrbier

**Affiliations:** 1 Queensland Institute of Medical Research, Brisbane, Queensland, Australia; 2 Department of Infectious Diseases, Alfred Hospital, Melbourne, Victoria, Australia; 3 Faculty of Medicine, University Malaya, Kuala Lumpur, Malaysia; 4 CBio Ltd., Brisbane, Queensland, Australia; 5 Monash University, Melbourne, Victoria, Australia; 6 Centre for Virology, Burnet Institute, Melbourne, Victoria, Australia; 7 School of Biomolecular and Physical Sciences, Griffith University, Brisbane, Queensland, Australia; University of Nebraska Medical Center, United States of America

## Abstract

Circulating heat shock protein 60 (Hsp60) and heat shock protein 10 (Hsp10) have been associated with pro- and anti-inflammatory activity, respectively. To determine whether these heat shock proteins might be associated with the immune activation seen in HIV-infected patients, the plasma levels of Hsp60 and Hsp10 were determined in a cohort of 20 HIV-infected patients before and after effective combination anti-retroviral therapy (cART). We show for the first time that circulating Hsp60 levels are elevated in HIV-infected patients, with levels significantly reduced after cART, but still higher than those in HIV-negative individuals. Hsp60 levels correlated significantly with viral load, CD4 counts, and circulating soluble CD14 and lipopolysaccharide levels. No differences or correlations were seen for Hsp10 levels. Elevated circulating Hsp60 may contribute to the immune dysfunction and non-AIDS clinical events seen in HIV-infected patients.

## Introduction

In mammals heat shock protein 60 (Hsp60) and heat shock protein 10 (Hsp10) are found in the mitochondria where they assemble into two back-to-back heptameric Hsp60/Hsp10 rings that function as ATP-dependent protein folding machines [1], [2]. Both Hsp60 and Hsp10 are also found in the circulation, where they appear to be associated with pro-inflammatory [3], [4], [5] and anti-inflammatory [2] activities, respectively. Elevated levels of circulating Hsp60 have been associated with a number of disease states including type 2 diabetes [6], hepatitis B [7], cardiovascular disease [8], atherosclerosis [4], [9], colorectal cancer [10], periodontitis [11] and juvenile idiopathic arthritis [12], with circulating levels also reported to be remarkably constant over time [13].

Herein we describe an analysis of the levels of circulating Hsp60 in HIV-infected patients before and after suppressive combination antiretroviral therapy (cART). We undertook this analysis because (i) translocation of bacterial products, such as lipopolysaccharide (LPS), into the circulation is a key cause of chronic immune activation in HIV patients [14], [15] and extracellular Hsp60 has been reported to augment inflammation via Toll like receptor 4 (TLR4), the receptor for LPS [5], [16], and (ii) increased apoptosis has been observed in HIV-infected patients on or off cART [17], with extracellular Hsp60 reported to promote apoptosis, again via TLR4 [18], [19], [20]. Whether Hsp10 interacts with Hsp60 in the extracellular milieu is unknown, but such an interaction has been suggested as a potential mechanism of action of XToll® [1]. XToll® is an Hsp10-based injectable biological drug being developed as an immunomodulatory agent, primarily for treatment of inflammatory autoimmune diseases [2], [21]. The circulating levels of Hsp10 were thus also analyzed.

**Figure 1:**
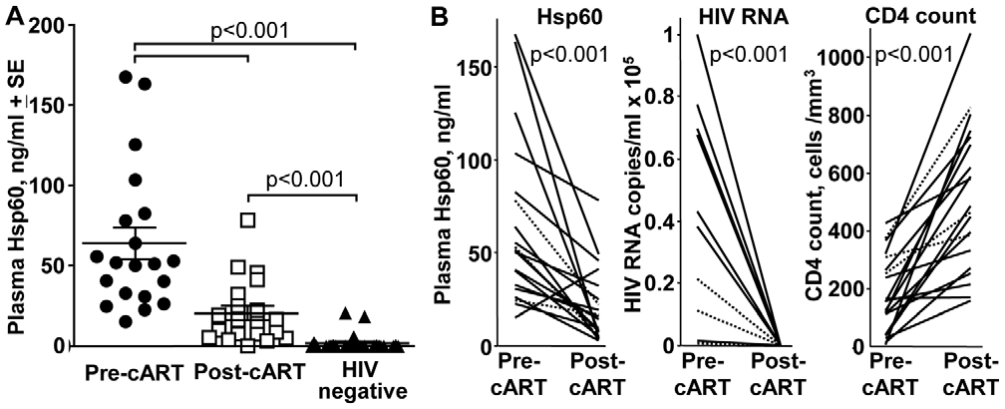
Circulating Hsp60. (A) Hsp60 levels, as measured by Hsp60 ELISA Kit (Stressgen, Ann Arbor, MI, USA), are shown for plasma samples from 20 HIV-infected patients before and after cART, and for plasma samples from 25 individuals seeking non-occupational post-exposure prophylaxis who emerged to be HIV negative. For cohort details see Table S1. Statistical comparisons between groups by the non-parametric Kolmogorov-Smirnov test. (B) Hsp60 – plasma levels of Hsp60 before and after cART (the same data as shown in A), with data from each patient treated as a matched pair and represented by a single line. HIV RNA – HIV RNA copies per ml before and after cART, with data from each patient treated as a matched pair. CD4 count – CD4 counts before and after cART, with data from each patient treated as a matched pair. Dotted lines represent patients who were also seropositive for Hepatitis C. Statistical analysis used the non-parametric Wilcoxon matched-pair signed-rank test.

## Materials and Methods

### Patient and control samples

Plasma samples from HIV-infected patients (n = 20) were collected as part of a previously reported study [14]. The study protocol was approved by the ethics committee of Alfred Health, and all patients provided written informed consent (Melbourne, Asutralia). The plasma samples were taken before, and (a mean of 1353± SD 918 days) after administration of cART. The mean age of the patients was 44± SD 11 years at the time of cART initiation, and all but 1 participant was male. Three patients were also hepatitis C antibody positive, and none were positive for hepatitis B surface antigen (HBsAg). HIV RNA copies, CD4^+^ T cell counts, plasma LPS and circulating soluble CD14 (sCD14) levels were also determined, as described previously [14]. Plasma samples collected from 25 individuals seeking non-occupational post-exposure prophylaxis, who emerged to be HIV negative, were also tested for circulating Hsp60 levels. Commercially available serum samples from HIV negative individuals were also tested for Hsp10 levels. Full details of all samples are provided in Table S1.

**Figure 2:**
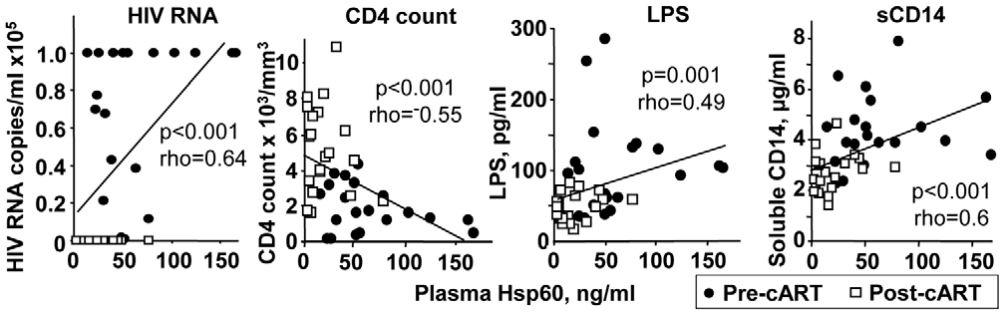
Hsp60 correlations with biomarkers. Correlations between plasma Hsp60 levels and plasma viral loads (HIV RNA), blood CD4 counts (CD4 count), plasma LPS levels (LPS) and plasma soluble CD14 levels (sCD14). Statistical analysis was performed using Spearman's rank correlation test; both p values and Spearman rank correlation coefficients (rho) are shown.

### Capture ELISA tests for Hsp60 and Hsp10

Plasma samples were collected in standard EDTA tubes, spun at 1000 g for 15 mins and supernatants aliquoted and stored at −80°C. Thawed samples were diluted 1 in 5 in PBS prior to analysis. Hsp60 levels were determined using the Hsp60 ELISA kit from Stressgen (Ann Arbor, MI, USA); the detection limit was 0.1 ng/ml of plasma. Circulating Hsp10 levels were measured using a sandwich ELISA, developed and optimised in-house. The assay used anti-Hsp10 goat polyclonal antibody (R&D Systems, Minneapolis, MN, USA) as both the immobilised capture and detection antibodies, and was optimised and validated by spiking plasma and serum with undetectable Hsp10 levels with recombinant Hsp10 [1]. The assay had a detection limit of 0.5 ng of Hsp10 per ml for plasma and 0.4 ng/ml for serum (data not shown).

**Figure 3:**
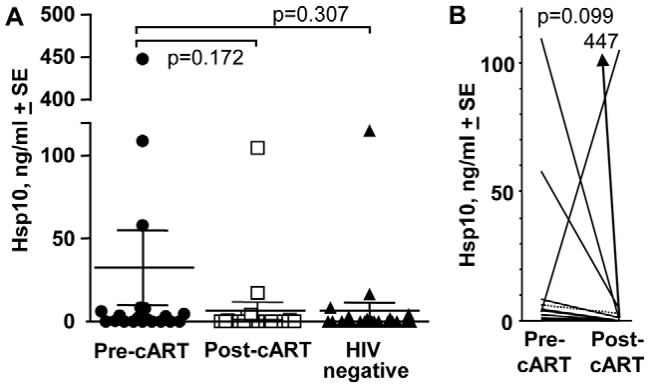
Circulating Hsp10. (A) Plasma Hsp10 levels in 20 HIV patients before and after cART, and Hsp10 levels in samples from 23 HIV-negative individuals, as measured by an in-house Hsp10 capture ELISA. Statistical comparisons between groups by Kolmogorov-Smirnov test. (HIV negative samples were obtained from both plasma and commercially available serum samples; Hsp10 levels were not significantly different in plasma and serum – Table S1). (B) Hsp10 levels (the same data as shown in A) with data from each patient treated as a matched pair and represented by a single line. Statistics by Wilcoxon matched-pair signed-rank test.

### Statistical analyses

Statistical analysis was performed using SPSS for Windows (version 19; SPSS, Chicago, IL, USA). Heat shock protein levels in separate groups were compared using the non-parametric Kolmogorov-Smirnov test; the data was not normally distributed and had large differences in variances. The non-parametric Wilcoxon matched-pair signed-rank test was used to compare heat shock protein levels before and after cART treatment with each patient providing a matched pair of data; the data was not normally distributed. Correlations were analysed using the non-parametric Spearman's rank correlation test, which provides a p value and a Spearman rank correlation coefficient (rho), which ranges from −1 (perfect negative correlation) to +1 (perfect positive correlation) with 0 denoting no correlation.

## Results

The plasma Hsp60 levels were determined in a cohort of HIV-infected patients before cART (pre-cART) and (1353± SD 918 days) after cART (post-cART) in the same patients. When the Hsp60 levels were compared (treating pre- and post-cART as independent groups), the plasma Hsp60 levels were significantly higher pre-cART, with both pre- and post-cART levels also significantly higher than those found in a group of HIV-negative individuals (Fig. 1A, Table S1) (see also below). When the pre- and post-cART Hsp60 levels were plotted for each patient (treating data for each patient as a matched pair), a significant difference between pre- and post-cART levels was again evident, with Hsp60 levels falling after cART in all but one patient (Fig. 1B, Hsp60). As expected following cART, HIV RNA levels were reduced to below detection (<50 RNA copies/ml) (Fig. 1B, HIV RNA) and the CD4 counts increased significantly (Fig. 1B, CD4 count) [14]. cART also resulted in a significant drop in circulating levels of sCD14 and LPS in these patients (Fig. S1A) [14].

Hsp60 levels in HIV-infected patients post-cART remained significantly higher than those found in the cohort of HIV-negative individuals, who were recruited following presentation for non-occupational post-exposure prophylaxis (Fig. 1A). The Hsp60 levels found in these HIV-negative individuals were comparable to those reported for healthy control individuals in a recent study [10]. The mean age of the HIV-negative cohort was 31.2 and the HIV-infected patients post-cART was 47.6 (Table S1), so this data was not derived from age matched cohorts. However, if the 10 youngest from the HIV-negative group and the 11 oldest from the post-cART group were removed to make the mean ages similar (and the same test as shown in Fig. 1A applied) significance was retained (p = 0.002, Kolmogorov-Smirnov test). In addition, circulating Hsp60 levels have been reported to decline with age [22], so age adjustment based on this report [22] would further increase the difference between HIV-negative and HIV-infected individuals.

When circulating Hsp60 levels were correlated with plasma HIV RNA (copies/ml), plasma LPS and plasma sCD14 levels, statistically significant positive correlations were evident in each case (Fig. 2, HIV RNA, LPS, sCD14). Hsp60 levels and CD4 counts also demonstrated a statistically significant negative correlation (Fig. 2, CD4 count). These results show for the first time that effective cART is associated with a reduction in the levels of circulating Hsp60 in HIV-infected patients, with Hsp60 levels also correlating with several major biomarkers of HIV infection. However, no significant correlations emerged if pre- and post-cART data were analyzed separately, indicating that the main effect was dependent on cART.

There were no significant differences in circulating Hsp10 levels in HIV-infected patients before and after cART, or between HIV-infected patients and samples from HIV-negative individuals (Fig. 3, Table S1). None of the correlations (shown in Fig. 2 for Hsp60 levels) between clinical biomarkers and Hsp10 levels were significant (data not shown). These observations suggest that circulating Hsp60 and Hsp10 levels are independently regulated in this setting. However, when all the available data (from HIV infected patients pre- and post-cART, and HIV-negative individuals) was used, a significant positive correlation between Hsp10 and Hsp60 levels did emerge (Fig. S1B), which perhaps indicates some level of co-regulation.

## Discussion

The higher levels of circulating Hsp60 in HIV-infected patients raise a number of questions regarding its source and activity. Hsp60 may be released from cells as a result (i) of viral infection, as reported for Hepatitis B infection of hepatocytes [7] and/or (ii) of translocation of Hsp60 to the cell surface as a result of apoptosis [18] and/or (iii) of active secretion in exosomes [23]. Current evidence suggests that circulating Hsp60 contributes to inflammation [3], [4], [5], a view perhaps consistent with the good correlation between Hsp60 levels and circulating levels of sCD14 (Fig. 2, sCD14), a biomarker of monocyte activation [24]. Given the association between elevated Hsp60 levels and cardiovascular disease in HIV-negative individuals [8], [9], the elevated Hsp60 levels in HIV-infected patients may contribute to the increased risk of cardiovascular disease seen in these patients [25]. Hsp60 has also been shown to increase apoptosis of osteoblasts *in vitro* [20] and to increase bone absorption in rats [26]. Elevated Hsp60 levels may thus also contribute to the increased risk of osteoporosis seen in HIV-infected patients [27]. The current study also suggests that circulating Hsp60 levels do not return to normal after cART (Fig. 1A). Elevated Hsp60 may thus also contribute to the ongoing immune dysfunction and/or non-AIDS clinical events (such as cardiovascular disease) in HIV-infected patients on cART [24]. However, whether circulating Hsp60 represents a clinically informative biomarker or even a viable target for therapeutic intervention remains to be explored.

## Supporting Information

Figure S1 (A) The change in circulating LPS and sCD14 levels between pre-cART and post-cART plasma samples, and (B) Spearman's rank correlation test comparing plasma Hsp10 levels with plasma Hsp60 levels using all available data.
(PDF)

Table S1 Clinical data and Hsp60 and Hsp10 levels for HIV-infected patients and HIV-negative individuals. This table provides all the data for each sample used in the study. The data was used to generate the figures and calculate statistical significances.
(PDF)
