# Supplementary material for: Circulating Heat Shock Protein 60 Levels Are Elevated in HIV Patients and Are Reduced by Anti-Retroviral Therapy
Source: PLoS One. 2012 Sep 28;7(9):e45291. doi: 10.1371/journal.pone.0045291 (PMC3460931; doi:10.1371/journal.pone.0045291)
Supplement: Figure S1 — (A) The change in circulating LPS and sCD14 levels between pre-cART and post-cART plasma samples, and (B) Spearman's rank correlation test comparing plasma Hsp10 levels with plasma Hsp60 levels using all available data. (PDF) [file pone.0045291.s001.pdf]

Supplemental Figure 1

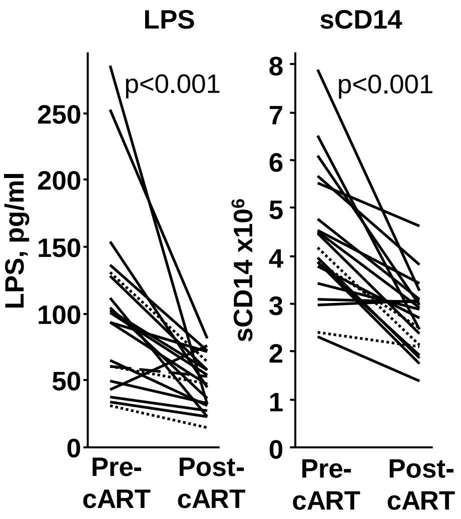

(A) The change in circulating LPS and sCD14 levels between Pre-cART and Post-cART plasma samples. Dotted lines represent patients also seropositive for Hepatitis C. Statistical differences between Pre-cART and Post-cART was calculated using the non-parametric Wilcoxon matched-pair signed-rank test.

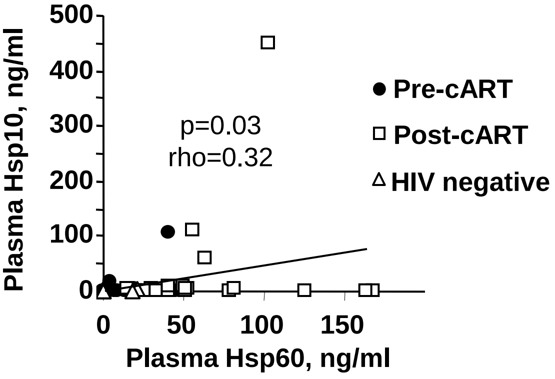

(B) Spearman's rank correlation test comparing plasma Hsp10 levels with plasma Hsp60 levels using all available data; 20 HIV patients pre- and post-cART and 6 HIV negative samples.
