# Supplementary material for: Circulating Heat Shock Protein 60 Levels Are Elevated in HIV Patients and Are Reduced by Anti-Retroviral Therapy
Source: PLoS One. 2012 Sep 28;7(9):e45291. doi: 10.1371/journal.pone.0045291 (PMC3460931; doi:10.1371/journal.pone.0045291)
Supplement: Table S1 — Clinical data and Hsp60 and Hsp10 levels for HIV-infected patients and HIV-negative individuals. This table provides all the data for each sample used in the study. The data was used to generate the figures and calculate statistical significances. (PDF) [file pone.0045291.s002.pdf]

Supplemental Table 1. Clinical data and Hsp60 and Hsp10 levels for HIV-infected patients and HIV-negative individuals

Patients	1	2	3	4	5	6	7	8	9	10	11	12	13	14	15	16	17	18	19	20	
**Sex**	Male	Male	Male	Male	Male	Male	Female	Male	Male	Male	Male	Male	Male	Male	Male	Male	Male	Male	Male	Male	Average age
**Age at cART initiation**	47	59	56	45	46	40	39	30	57	27	30	34	46	53	69	44	47	35	39	34	43.9
**Hepatitis B**	No	No	No	No	No	No	No	No	No	No	No	No	No	No	No	No	No	No	No	No	
**Hepatitis C**	No	No	No	No	No	yes	No	No	No	yes	No	No	yes	No	No	No	No	No	No	No	
**Pre-cART**																					
**LPS (pg/ml)**	49.73	127.92	285.49	102.21	37.5	31.23	34.05	111.81	153.74	60.06	92.89	100.17	131.08	43.06	136.08	93.48	252.81	64.6	103.95	60.23	
**sCD14 (pg/ml))**	3.79E+06	4.50E+06	6.09E+06	3.42E+06	2.97E+06	2.39E+06	2.30E+06	3.09E+06	4.78E+06	4.17E+06	3.96E+06	6.52E+06	3.86E+06	5.52E+06	7.89E+06	4.52E+06	3.87E+06	4.48E+06	5.66E+06	3.86E+06	
**CD4 (cells/ml)**	367	157	30	39	320	374	311	15	238	427	124	9	252	43	112	266	112	153	118	167	
**HIV RNA copies/ml**	100000	100000	100000	100000	1575	21200	77500	69500	43100	875	100000	100000	11300	100000	100000	100000	67700	100000	100000	38200	
**Hsp60 (ng/ml)**	40.63	103.5	51.75	167.5	50	30.63	24.63	22.38	40.25	52.88	125.5	26.125	78.13	55.5	82.5	15.25	32.75	51	163.25	63.88	
**Hsp10 (ng/ml)**	0	447.34	0	0	8.02	6.23	0	0	8.61	2.23	1.26	0	0	109	3.61	3.24	0.52	4.69	0	57.96	
**Post-cART**																					Average age
**Days post-cART**	389	995	1597	1862	2996	2223	1077	1412	836	3079	1463	3012	329	699	480	644	700	2157	530	581	47.6
**LPS (pg/ml)**	33.12	57.94	32.22	57.46	27.47	14.93	23.08	22.76	45.1	52.64	71.07	53.98	64.16	76.01	72.19	46.41	81	31.28	36.69	47.15	
**sCD14 (pg/ml))**	2.70E+06	2.91E+06	2.98E+06	2.87E+06	3.07E+06	2.09E+06	1.39E+06	3.04E+06	3.08E+06	2.13E+06	1.86E+06	2.45E+06	2.50E+06	4.63E+06	3.28E+06	3.42E+06	1.74E+06	2.38E+06	3.80E+06	1.92E+06	
**CD4 (cells/ml)**	725	216	160	449	1084	828	384	270	333	582	590	803	466	488	254	618	398	700	747	170	
**HIV RNA copies/ml**	<50	<50	<50	<50	<50	<50	<50	<50	<50	<50	<50	<50	<50	<50	<50	<50	<50	<50	<50	<50	
**Hsp60 (ng/ml)**	14.13	78.38	7.63	49.13	32.13	19.25	16.5	9.75	5.25	4.88	7.38	0	22.13	24.88	45.63	41.25	16.00	8.63	3.88	3.25	
**Hsp10 (ng/ml)**	0	1.14	0	0	1.01	2.82	0	0	17.34	0	0	0	0	0	0	104.85	0	0	0	4.37	

NA - not available

**HIV negative**	1	2	3	4	5	6	8	9	10	11	12	13	14	15	16	17	18	19	20	21	22	23	24	25	26	Average ag
**Age**	NA	NA	NA	NA	NA	NA	34	35	26	48	30	33	21	23	29	29	30	25	34	37	19	33	32	36	39	31.2
**Sex**	NA	NA	NA	NA	NA	NA	M	M	M	M	M	M	M	M	M	M	M	M	M	M	M	M	M	M	M	
**Hsp60 (ng/ml)**	20.75	0	0	0	0	18.63	0	0.36	2.40	0	2.36	0	0	0	0	6.02	0	0.10	0.15	0	0.53	0.27	0.57	0.27	0	

Serum samples from 1. Bioreclamtion. 2. Sigma-Aldrich. 3. Innovative Research. 4. Lonza

**HIV negative**	1	2	3	4	5	6	7	BRH177746[1]	BRH177752[1]	BRH177750[1]	BRH177749[1]	BRH177751[1]	BRH177754[1]	BRH177755[1]	BRH177748[1]	115K89091[2]	BRH177747[1]	45k0462[2]	E70801-10[illegible]	E70801-107[3]	E70801-121[3]	E70801-150[3]	L1116313[4]
**Hsp10 (ng/ml)**	4.20	0	3.07	0	0	0	16.54	1.58	0	115.08	0	0	0	8.62	0	1.92	0	0	0	0	0	0	0

ge
